# The impact of digital inclusive financial development on local government expenditure: Evidence from China

**DOI:** 10.1371/journal.pone.0300775

**Published:** 2024-05-16

**Authors:** Yuran Chen, Qian Huang, Qiaoyun Zhang

**Affiliations:** 1 School of Finance, Southwestern University of Finance and Economics, Chengdu, Sichuan, China; 2 School of Finance, Yunnan University of Finance and Economics, Kunming, Yunnan, China; University of Murcia: Universidad de Murcia, SPAIN

## Abstract

This paper investigates the impact of digital inclusive financial development on local government expenditure incentives at the income level. It does so by constructing a multi-level government Dynamic Stochastic General Equilibrium (DSGE) model that incorporates the financial sector. By employing empirical methods that involve uncertainty shocks and counterfactual simulations, the research yields several key findings. Firstly, the development of digital inclusive finance contributes to breaking down the urban-rural dual financial structure, thus facilitating balanced economic development within regions. Secondly, it reduces the proportion of financially excluded areas, accelerates fiscal decentralization, leading to an increase in local government fiscal revenue, and, consequently, an expansion of local fiscal expenditures. Thirdly, at a certain stage of digital inclusive finance development, it tends to crowd out residents’ investment and consumption. Therefore, the decentralization of fiscal power and the expansion of local government expenditure at this stage may paradoxically inhibit regional economic growth. The study’s conclusions validate the significant impact of digital inclusive finance on local government incentives at the income level.

## 1 Introduction

Against the backdrop of globalization, fostering the efficient and collaborative development of fiscal and financial systems worldwide has become a crucial pillar for driving high-quality economic development [1]. As the lifeblood of socio-economic advancement, finance plays a pivotal role in enhancing and consolidating the sustainability of government fiscal operations. It provides a solid empirical foundation for nations to achieve their established objectives. Existing research, both domestically and internationally, on fiscal and financial matters often approaches the subject from the perspective of fiscal expenditure. It predominantly explores how finance underpins government fiscal spending through local government debt, effectively serving as a second line of defense [2–4], and the first line of defense refers to balancing fiscal expenditures with local government budgetary revenues. However, there is limited scholarly exploration from the perspective of fiscal revenue to ascertain whether financial sector development also influences local fiscal behavior. Moreover, with the accelerated pace of global digitization empowering the development of inclusive finance, financial development has deepened the breadth and depth of financial coverage globally.

Thus, under the backdrop of digitalization, the impact of finance on the expenditure behavior of local governments in various countries remains inadequately addressed in existing literature. Therefore, this study examines the relationship between fiscal and financial elements from the fiscal revenue perspective, presenting a novel approach to advancing the coordinated development of fiscal and financial systems.

Research on the development of digital inclusive finance and the expenditure behavior of local governments in China carries significant implications. This is especially pertinent given recent developments, where the standardized and efficient growth of the traditional financial sector in China has injected new vitality into local economies, leading to an increase in local government fiscal capacity. The specific pathway manifests as follows: the traditional financial sector propels regional economic growth by utilizing channels such as households, industry systems, and government departments. The dividends of economic growth further contribute to the overall increase in the fiscal capacity of local governments. Additionally, considering that many tax-paying enterprises in China are located below the provincial level, the prevailing fiscal decentralization reform in China has made tax-sharing rules a key component of fiscal revenue decentralization [5]. However, under the current tax distribution system, there may be a mechanism path of fiscal revenue decentralization, represented by tax-sharing, leading to an expansion of the scale of local government fiscal expenditures. Furthermore, influenced by the practical challenges of weak financial infrastructure in remote areas and the credit gaps among rural residents, financial exclusion in economically disadvantaged regions, such as rural areas, has been a persistent issue.

However, the rapid development of digital technology in China has overcome the “bottlenecks” of traditional financial growth, opening up possibilities for achieving inclusive growth goals and reshaping the fiscal allocation pattern of local governments. Specifically, modern information technologies such as big data and cloud computing can construct precise credit profiles for transaction entities, effectively alleviating information asymmetry between the financial sector and demand entities. This can reduce information frictions and mitigate market failures. In the process of inclusive finance promoting financial poverty alleviation, the development paradigm of “digital technology + inclusive finance” plays a role in lowering the threshold of financial services and reducing the costs of financial services. It represents a new model for achieving fast, efficient, low-cost, and wide-reaching financial services. This model not only helps boost rural economic development, elevate the overall fiscal revenue level of localities, and ensure the sustainability of government fiscal expenditures, but it also effectively expands the depth and breadth of financial services for small and medium-sized enterprises as well as urban and rural residents. This, in turn, facilitates the realization of inclusive growth goals.

In summary, it is evident that the sustained development of modern finance needs to encompass both urban and rural dimensions. There is a pressing need to expedite digitization efforts that broaden and deepen the impact of inclusive finance. This acceleration is pivotal in aiding fiscal governance functions to achieve the objectives of inclusive growth. The potential marginal contributions of this paper lie in two aspects. Firstly, by examining the impact mechanism of the development of digital inclusive finance on local government fiscal behavior from the fiscal revenue perspective, the study contributes to enhancing and supplementing research on the coordinated development of fiscal and financial systems from multiple perspectives, both domestically and internationally. Secondly, the findings of this study can serve as a valuable reference and support for countries worldwide seeking to achieve the coordinated development of fiscal and financial systems through the development of digital inclusive finance.

The structural framework ahead is delineated as follows: commencing with a comprehensive literature review and critique, followed by an exploration of fundamental facts and theoretical hypotheses. Subsequently, the focus shifts towards elucidating the intricacies of model design and parameter calibration, paving the way for dynamic simulation analyses. Finally, the culmination of this framework involves presenting the findings gleaned from these analyses, accompanied by actionable recommendations.

## 2 Literature review

This paper focuses on the impact of the development of digital inclusive finance on the expenditure behavior of local governments from the perspective of fiscal revenue. However, existing literature has not directly explored the relationship between the two. Instead, it predominantly concentrates on the relationship among financial development, fiscal decentralization, and the fiscal behavior of local governments from the perspective of fiscal expenditure.

Firstly, there is a fundamental consensus that the development of digital inclusive finance can effectively expand traditional financial boundaries, mitigate financial exclusion, thereby narrowing the urban-rural income gap, and achieving inclusive growth [6]. Jack et al. (2013) posit that the advancement of digital finance alleviates the “wealth threshold” effect in traditional financial markets, enhances financial market efficiency, promotes price discovery and information circulation, improves channels for impoverished individuals to access credit and savings, and contributes to the increased inclusivity of financial transfers and services [7, 8]. Simionescu and Cifuentes-Faura (2023) examine the main drivers of per capita debt in 32 Mexican states over the period 2006–2021. The results of the study show that poverty is identified as the main determinant of debt [9]. Moreover, digital inclusive finance, through income and growth effects, can influence entrepreneurial activities within households, leading to increased household income [10].

Secondly, from a fiscal perspective, Molotok (2020) and Gillman (2021) define the ratio of tax sharing between provincial and prefecture-level cities as a crucial indicator for assessing the distribution of fiscal power among governments below the provincial level [11, 12]. After systematically organizing and summarizing the tax-sharing situations within provincial-level local governments, Jin and Rider (2022) found significant variations in tax-sharing rules among different provinces [13]. However, the majority of provinces still employ an “incremental sharing” approach to stimulate economic development at the city and county levels within their jurisdictions, gradually devolving fiscal powers to promote economic growth. Cifuentes-Faura et al. (2023) argue that local autonomy provides local entities with the freedom to manage their own interests within the legal framework of the State [14]. From the standpoint of local financial development, financial growth facilitates “intensive” economic growth at the local level. However, within the fiscal decentralization system, local government intervention in directing financial funds also supports an “extensive” style of economic growth [15, 16].

Thirdly, research on the impact of fiscal decentralization on the size of government expenditures has garnered attention. Scholars, drawing on classical public economics theories, contend that fiscal decentralization enhances the efficiency of local fiscal expenditures, thereby effectively controlling local public spending [17, 18]. According to the “common pool theory,” an excessive reliance of local governments on national “common pool” resources may lead to an expansion of government expenditures when fiscal decentralization is in place [19]. Rodden (2002), approaching the issue from the perspective of government organizational structure, posits that fiscal expenditure and revenue decentralization exert different influences on the size of local government expenditures. Fiscal expenditure decentralization is negatively correlated with the size of local government expenditures, while fiscal revenue decentralization demonstrates a positive correlation [20]. Compared to the central government providing standardized products and services to different regions, local governments can reduce the cost of public services, enhance fiscal expenditure efficiency, and exercise control over shared taxes to promote local fiscal stability and mitigate fiscal imbalances [21, 22].

Fiscal decentralization endows local officials with increased autonomy in economic and administrative decision making. This empowerment enables them to utilize public policy instruments such as subsidies and tax reductions to assist businesses in enhancing production efficiency. Consequently, a sustainable mechanism for local economic growth is established at the microeconomic level. Moreover, the fiscal decentralization system in China contributes to reinforcing intergovernmental competition, effectively constraining the behavior of local governments [23]. Cifuentes-Faura et al. (2023) have demonstrated that municipalities are more efficient when there is greater economic and financial transparency, coupled with more information available on public service contracts, urban planning, and public works. This enables more optimal management of the scarce resources of public administrations [24]. Wu et al. (2023) posit that fiscal revenue decentralization tends to shift toward sub-provincial local governments as regional stability increases. This shift is primarily driven by the fact that in regions with more stable economic development, the provincial government’s need to control transfer payments to stabilize the overall economy diminishes. This reduction, in turn, increases the proportion of tax revenue sharing at the municipal and county levels, aiming to incentivize their development [25].

In summary, research on the synergistic relationship between finance and fiscal matters has predominantly focused on expenditure, investigating the interplay among financial development, fiscal decentralization, and local government fiscal behavior. Fewer studies have approached the issue from the revenue perspective, exploring the synergistic relationship between finance and fiscal matters. Therefore, this paper aims to address this gap by situating the problem within a new DSGE model that encompasses a microeconomic foundation involving individuals, businesses, and government entities. Leveraging a dual perspective on financial development and fiscal decentralization, the study will integrate both angles and incorporate absolute numerical values for stochastic simulations to validate traditional financial impact mechanism pathways. Building upon this foundation, further insights will be gained through counterfactual simulations to explore how digital inclusive finance influences changes in local government behavior patterns.

## 3 Fundamental facts and theoretical hypotheses

### 3.1 Fundamental realities of China’s rural-urban dualistic economic structure differentiation

China has experienced persistent rural-urban dualism in its economic development. In the early years of the People’s Republic of China, the national development strategy primarily prioritized the vigorous development of heavy industries. This strategic emphasis aimed to enable the country to independently confront external threats. Consequently, during this period, rural financial development had to align with the overall national economic development strategy.

From the inception of the reform and opening-up era until the early 21st century, China implemented a series of reforms in its rural areas. These reforms were conducted within the existing national economic system and achieved a certain degree of success. However, as the process of advancing market-oriented economic reforms continued, rural financial reform gradually transformed into financial reforms oriented towards urban and industrial development. The government attempted to guide fund flows through financial development, directing funds into the state-owned economy.

Table 1 reports the trends of urban-rural financial usage breadth, usage depth, and binary differentiation calculated using the entropy weight method in China. It is important to note, firstly, that the Peking University Digital Finance Research Center began publishing the Urban Inclusive Digital Financial Coverage Index in 2012 and the Rural Inclusive Digital Financial Coverage Index in 2014. Therefore, the author selected data from around 2014, spanning five years, for the analysis. Secondly, drawing inspiration from Hasan et al.’s (2022) quantitative criteria for financial dualism, this study extended the methodology to additional years. It utilized the proportions of urban financial loans, agricultural financial loans, urban network points, and rural network points, respectively, in relation to the total loans and network points at the local level to represent the depth and breadth of financial usage [26]. Thirdly, following the methodology outlined in the “Peking University Inclusive Digital Financial Index Report 2011–2020,” which employed nighttime light data and a 1-kilometer grid map to demarcate urban and rural areas, this study introduced the Digital Inclusive Financial Index. The entropy weight method was then employed to calculate the trends in China’s urban-rural financial dualism [27]. In terms of usage breadth, traditional financial coverage is approximately 60% in urban areas and around 40% in rural areas. Over time, the urban-rural differentiation trend in traditional finance has gradually stabilized. Additionally, it is evident that since 2012, digitization has accelerated the diffusion and development of the financial industry in urban and rural China. Digital inclusive financial services have progressively extended to remote rural areas, overcoming bottlenecks in traditional financial development. The indices for urban and rural digital inclusive financial coverage have both significantly increased at a rapid pace.

**Table 1:** Specific measurement indicators for urban-rural financial dual differentiation.

		2009	2012	2014	2019
Using breadth	urban traditional financial coverage (%)	58.97	61.78	62.58	63.73
	urban bank branches quantity (10000 units)	11.14	12.45	13.40	14.44
	rural traditional financial coverage (%)	41.03	38.20	37.42	36.27
	rural bank branches quantity (10000 units)	7.74	7.73	8.01	8.22
	urban digital inclusive financial coverage index	\	86.70	148.93	238.92
	rural digital inclusive financial coverage index	\	\	49.03	95.96
Using depth	urban financial loan percentage (%)	94.56	73.99	73.14	77.34
	urban financial loan quantity (trillion CNY)	37.83	49.49	63.42	119.19
	agricultural financial loan percentage (%)	5.44	26.01	26.86	22.66
	agricultural financial loan quantity (trillion CNY)	2.18	17.40	23.29	34.91
The urban-rural financial divergence trend		42.46	27.09	37.23	60.95

Note: The data utilized in this study was compiled by the author.

On the aspect of usage depth, in 2009, urban financial loans in China accounted for 94.56%, with agricultural financial loans representing 5.44%. In 2019, urban financial loans in China accounted for 77.34%, and agricultural financial loans represented 22.66%. These data indicate that with the rural development of digital inclusive finance, the financial coverage and the proportion of agricultural loans in China’s rural areas have continuously improved.

In summary, the development of digital inclusive finance not only extends financial coverage to remote rural areas but also enhances the depth of financial service usage among rural residents. However, when examining overall differentiation metrics, there persists a “scissors gap” style development in China’s urban and rural financial landscape, indicating a sustained dualistic pattern of financial differentiation between urban and rural areas.

### 3.2 Theoretical analysis and research hypotheses

**Fig 1:**
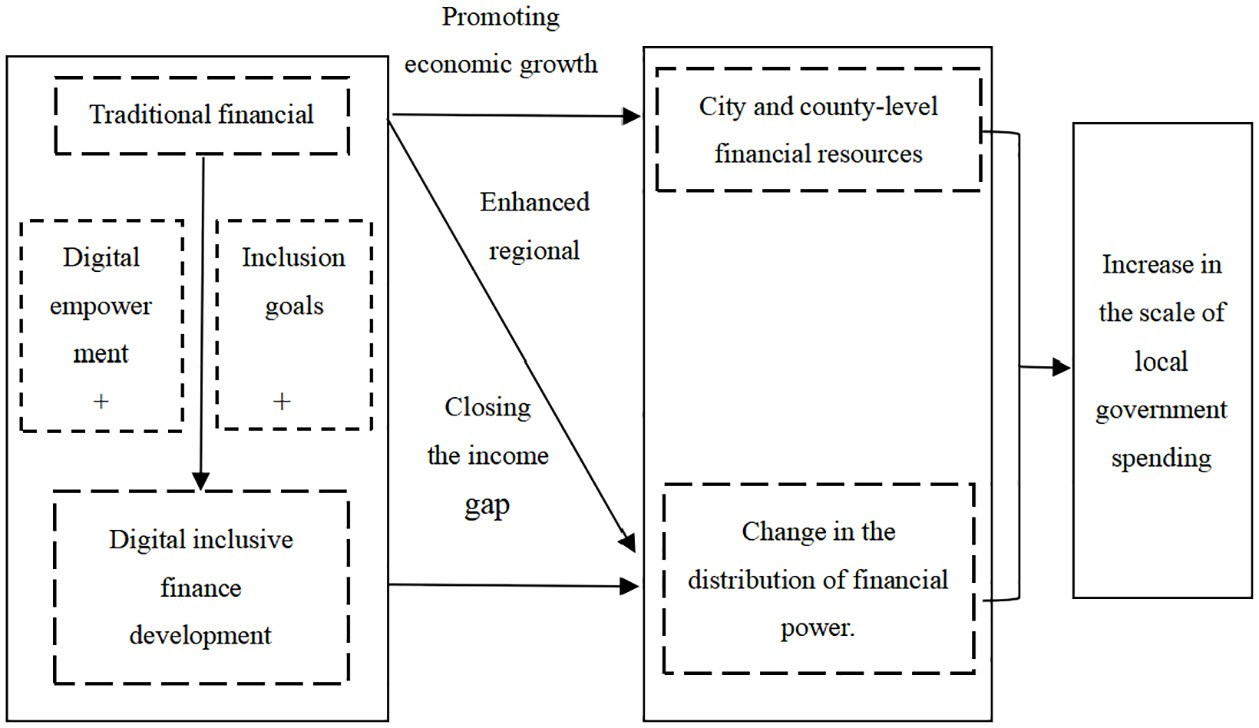
Mechanism road map.

Against the backdrop of dual differentiation in rural and urban finance in China, this study initiates its inquiry from the contemporary realities of both traditional financial development and the unfolding landscape of digital inclusive finance. Employing a fiscal decentralization perspective, exemplified by the segmentation of tax revenue, the research delves into the impact mechanisms and pathways of sub-provincial financial industry development on government behavior patterns in China. Fig 1 presents a specific schematic representation of these pathways. The specific mechanisms and pathways are summarized as follows:

The development of traditional financial services can contribute to meeting the financing needs of small and medium-sized enterprises, while also enhancing residents’ consumption and investment capacities, thereby stimulating local economic development. The relatively fixed rules of tax revenue allocation and incentive mechanisms can result in an increase in local fiscal revenues, with tax revenue serving as a representative indicator. In other words, as local government fiscal revenues increase, in tandem with the expanding responsibilities and fiscal expenditures at the local level, there is a propensity for an enlargement of local government expenditure. Simultaneously, discounting the impact of tax revenue allocation, the balance of economic development at the municipal and county levels below the provincial tier also influences fiscal decentralization, leading to an expansion in government expenditure [28]. Specifically, more balanced economic development at the municipal and county levels implies a decrease in the fiscal funds required by the provincial government to balance its economic development. Consequently, at the provincial level, there is a tendency to weaken fiscal fund control, delegate certain fiscal powers, and, upon the acquisition of fiscal autonomy by municipal and county governments, witness an increase in local government expenditure.

The impact of traditional finance on the fiscal decentralization and expenditure scale of local governments at the municipal and county levels is primarily evident in two aspects. Firstly, traditional finance facilitates local economic development. Under the prevalent tax revenue allocation system of “incremental sharing,” fiscal autonomy gradually shifts to the local level. This enhances the fiscal discretionary capacity of municipal and county-level governments, ultimately resulting in increased local government expenditure. Secondly, traditional finance promotes regional economic development by reducing income disparities and fostering balance in regional economic development. This dynamic results in decreased control exerted by provincial governments over fiscal funds, leading to the devolution of fiscal resources to the municipal and county levels. This decentralization aims to seek economic growth opportunities from development at the municipal and county levels, thereby driving regional economic growth [29]. Therefore, the following hypothesis is proposed:

H1: The development of traditional finance is suggested to lead to an increase in the scale of local government expenditure.

The rapid advancement of digital technologies has provided the traditional financial industry with innovative ways to effectively achieve the inclusive finance. Consequently, it influences and deepens the transformation of behavior patterns within local governments. In particular, digital inclusive finance, as a representative example of the paradigm shift in digital economic development, primarily focuses on underserved segments. This includes small and medium-sized enterprises with substantial capital needs but limited access to sufficient credit funds from traditional banks, as well as residents in remote rural areas with scarce collateral assets and no credit history. Furthermore, due to the virtual, replicable, and cost-effective nature of digital finance, constructing virtual financial service platforms incurs lower costs. This significantly enhances the reach of financial services to remote areas. As a result, digital inclusive finance accelerates financial service coverage in remote rural areas, contributing to the achievement of regional economic balance. This emphasizes the role of the financial sector, which leads to a relaxation of budget constraints and an increase in government expenditure. Therefore, the following hypothesis is proposed:

H2a: Digital inclusive finance is expected to promote inclusive growth, leading to an additional devolution of fiscal autonomy for governments.
H2b: The devolution of fiscal powers to the provincial-level government, resulting from the development of digital inclusive finance, is expected to ultimately stimulate a further increase in the scale of local government expenditures.

Therefore, this study explores the mechanisms and pathways of changes in local government behavior patterns under traditional and digital inclusive finance backgrounds. It achieves this by designing and constructing a multi-level government DSGE model that incorporates the financial sector. The research employs uncertainty-driven stochastic shocks and counterfactual simulations to validate the theoretical hypotheses outlined earlier.

## 4 Model design and parameter calibration

### 4.1 Model design

#### 4.1.1 Urban area

Given the relatively developed financial market and the absence of financial exclusion in the urban area, widespread financial services enable consumers to make intertemporal consumption and investment choices through the financial market, optimizing their individual preferences. At the same time, the financial sector in the urban area engages in optimal asset allocation decisions based on consumers’ savings and infinite-period discounted income. Regarding expenditures, consumers in the urban area can participate in consumption and private investment activities [30, 31]. Through savings, they can maximize their current period discounted utility. Therefore, the behavioral choice equation for consumers is formulated as follows:

$$\begin{array}{c} {\text{MaxE}}_{t}\sum_{i=1}^{\infty }\beta^{t}(\frac{c_{t}^{1-\xi }}{1-\xi }-\frac{L_{t}^{1+\varphi }}{1+\varphi }) \end{array}$$ (1)

$$\begin{array}{c} \;\text{s.t.}\;C_{t}^{1}+S_{t}^{1}+I_{t}^{1}=(1-\tau_{t}^{s})(W_{t}^{1}L_{t}^{1}+r_{t}^{k}K_{t-1}^{1}+r_{t-1}S_{t-1}^{1}) \end{array}$$ (2)

$$\begin{array}{c} K_{t}^{1}=K_{t-1}^{1}\left(1-\delta \right)+I_{t} \end{array}$$ (3)

The first-order conditions and Euler equations for relevant variables can be derived as follows:

$$\begin{array}{c} W_{t}^{1}=\frac{{\left(L_{t}^{1}\right)}^{\varphi }}{{\left(C_{t}^{1}\right)}^{-\zeta }\left(1-\tau_{t}^{1}\right)} \end{array}$$ (4)

$$\begin{array}{c} {\left(C_{t}^{1}\right)}^{-\xi }=\beta {\left(C_{t+1}^{1}\right)}^{-\xi }[(1-\tau_{t+1}^{k})r_{t+1}^{k}+\left(1-\delta \right)] \end{array}$$ (5)

When considering firms in the urban area, they absorb local labor and capital investments while benefiting from public capital investments by either the provincial or municipal governments. These investments may take the form of tangible tax incentives or positive externalities arising from public infrastructure development. Additionally, assuming a split-weighting ratio, denoted as the allocation between provincial and local governments, and a production elasticity of public capital, which represents the firm’s decision-making in an urban area, the formulation is as follows:

$$\begin{array}{c} \text{Max}\;(1-\tau_{t}^{q})Y_{t}^{1}P_{t}^{1}-(1+\tau_{t}^{1})W_{t}^{1}L_{t}^{1}-(1+\tau_{t}^{k})r_{t}^{k}K_{t}^{1} \end{array}$$ (6)

$$\begin{array}{c} \;\text{s.t.}\;Y_{t}^{1}=A_{t}^{1}K_{t-1}^{1-\alpha }{\left(1-L_{t}^{1}\right)}^{1-\alpha }{\left(G_{t}^{1}\right)}^{\chi } \end{array}$$ (7)

$$\begin{array}{c} {\text{G}}_{t}^{1}=g_{t}^{1\phi }g_{t}^{1-\phi } \end{array}$$ (8)

In Eq (8), $g_{t}^{1}$ represents the local municipal government’s proprietary public investment, while *g*_*t*_ denotes the provincial government’s public investment in the region. Consequently, the first-order conditions for the relevant variables can be derived as follows:

$$\begin{array}{c} W_{t}^{1}=\left(1-\alpha \right)(1-\tau_{t}^{q})Y_{t}^{1}P_{t}^{1}/(L_{t}^{1}-1) \end{array}$$ (9)

$$\begin{array}{c} r_{t}^{k}=\alpha (1-\tau_{t}^{q})Y_{t}^{1}P_{t}/K_{t-1}^{1} \end{array}$$ (10)

When considering the banking sector in the urban area, it participates in borrowing and lending with both the private and public sectors using resident savings and the net assets of the banking sector. In simpler terms, funds from the banking sector enter the market through private investments and local government bonds. The banking sector, subject to the previously mentioned budget balance conditions, maximizes its returns based on intertemporal discounted net assets. Thus, the decision making equation for the banking sector in the urban area can be formulated as follows:

$$\begin{array}{c} V_{t}^{1}={\text{MaxE}}_{t}\sum_{i=1}^{\infty }\left(1-\theta \right)\theta^{i}\beta^{i+1}N_{t+1+i}^{1} \end{array}$$ (11)

$$\begin{array}{c} \;\text{s.t.}\;N_{t}^{1}=(r_{t}^{k}-r_{t-1})K_{t-1}^{1}+(r_{t}^{b}-r_{t-1})B_{t-1}+r_{t-1}N_{t-1}^{1} \end{array}$$ (12)

$$\begin{array}{c} V_{t}^{1}⩾\lambda_{1}K_{t}^{1}+\lambda_{2}B_{t}^{1} \end{array}$$ (13)

Here, λ_1_ and λ_2_ represent the banking sector’s credit ratios to the private and public sectors, respectively. Because of the recursive relationship in the constraint equation for banking net assets, the solution requires an iterative approach, obtained through a speculative process, as outlined below:

We ensure the validity of Eq (14), then taking the first-order derivatives of Eq (14) yields Eqs (15) to (18).

$$\begin{array}{c} V_{t}^{1}=X_{t}^{1}K_{t}^{1}+X_{t}^{2}B_{t}^{1}+X_{t}^{3}N_{t}^{3} \end{array}$$ (14)

$$\begin{array}{c} X_{t}^{1}=\lambda_{1}\frac{\mu_{T}}{1+\mu_{T}}, \end{array}$$ (15)

$$\begin{array}{c} X_{t}^{2}=\lambda_{t}^{2}\frac{\mu_{T}}{1+\mu_{T}} \end{array}$$ (16)

$$\begin{array}{c} X_{t}^{2}=\lambda_{t}^{2}\frac{\mu_{T}}{1+\mu_{T}}, \end{array}$$ (17)

$$\begin{array}{c} K_{t}^{1}=\frac{X_{t}^{2}-\lambda_{t}^{2}}{\lambda_{t}^{1}-X_{t}^{1}}B_{t}^{1}+\frac{X_{t}^{3}}{\lambda_{t}^{1}-X_{t}^{1}}N_{t}^{1} \end{array}$$ (18)

By expressing the total value of the bank as a function of net assets in Eq (19), we can derive the corresponding speculative results, namely Eqs (20) to (23).

$$\begin{array}{c} V_{t}^{1}=(1+\mu_{t})X_{t}^{3}N_{t}^{1} \end{array}$$ (19)

$$\begin{array}{c} X_{t}^{1}=\beta o_{t+1}(r_{t+1}^{k}-r_{t}) \end{array}$$ (20)

$$\begin{array}{c} X_{t}^{2}=\beta o_{t+1}(r_{t+1}^{b}-r_{b}) \end{array}$$ (21)

$$\begin{array}{c} X_{t}^{3}=\beta o_{t+1}r_{t} \end{array}$$ (22)

$$\begin{array}{c} 0_{t}=(1-\theta +\theta (1+\mu_{t})X_{t}^{3}) \end{array}$$ (23)

Simultaneously, define the leverage of private credit and public credit as given in Eq (24), and define the overall financial leverage of the market as expressed in Eq (25).

$$\begin{array}{c} \Psi_{t}=\frac{B_{t}^{1}}{K_{t}^{1}} \end{array}$$ (24)

$$\begin{array}{c} \phi_{t}=\frac{K_{t}^{1}+B_{t}^{1}}{N_{t}^{1}}=\frac{(1+\psi_{t})X_{t}^{3}}{\left(1+\frac{\lambda_{1}}{\lambda_{2}}\psi_{t})(\lambda_{1}-X_{t}^{1}\right)} \end{array}$$ (25)

When considering the government in the urban area, its primary responsibility is to balance local fiscal revenues and expenditures. Therefore, the behavior of the government in the urban area can be encapsulated by the following budget constraint:

$$\begin{array}{c} g_{t}^{1}+r_{t}^{b}B_{t-1}^{1}=B_{t}^{1}+\Gamma_{t}T_{t}^{1} \end{array}$$ (26)

Here, $T_{t}^{1}$ represents the total tax revenue obtained by urban area through personal income tax, corporate income tax, value-added tax on capital elements, and value-added tax on labor elements. Γ denotes the tax revenue-sharing rules stipulated by the provincial government for urban area. Since the determination of the revenue-sharing ratio is often influenced only by actual variables, it is sufficient to consider the real changes in the corresponding macroeconomic variables. Additionally, let’s assume a price level of 1.

#### 4.1.2 Rural area

Given the presence of financial exclusion in the rural area, residents in this region experience lower income levels, limiting their ability to use financial instruments for intertemporal decision making. As a result, their decisions are constrained to maximizing utility in the current period. Similarly, firms and government departments in rural area engage in production and tax collection in the same manner as in rural area.

Their optimization behavior involves allocating resources between consumption and private direct investment in the current period to achieve maximized discounted utility, taking into account both labor income and capital gains. Therefore, their behavioral equation can be expressed as:

$$\begin{array}{c} {\text{MaxE}}_{t}\sum_{i=1}^{\infty }\beta^{t}(\frac{C_{t}^{2(1-\xi )}}{1-\xi }-\frac{L_{t}^{2(1+\varphi )}}{1+\varphi }) \end{array}$$ (27)

$$\begin{array}{c} \text{s.t.}\;C_{t}^{2}=(1-\tau_{t}^{1})W_{t}^{2}L_{t}^{2}+\pi_{t}^{2} \end{array}$$ (28)

Similarly, firms in rural area engage in production within the market. However, due to the inability of private individuals to invest, firms in this region rely solely on existing factor endowments and labour for production. Their behavioral equation can be expressed as:

$$\begin{array}{c} \text{Max}(1-\tau_{t}^{q})Y_{t}^{2}P_{t}^{2}-(1+\tau_{t}^{1})W_{t}^{2}L_{t}^{2} \end{array}$$ (29)

$$\begin{array}{c} \;\text{s.t.}\;Y_{t}^{2}=A_{t}^{2}{\left(1-L_{t}^{2}\right)}^{1-\chi }{\left(G_{t}^{2}\right)}^{\chi } \end{array}$$ (30)

$$\begin{array}{c} G_{t}^{2}=g_{t}^{2\phi }g_{t}^{1-\phi } \end{array}$$ (31)

In Eq (31), $g_{t}^{2}$ represents the local municipal government’s own public investment, and g_*t*_ denotes the provincial government’s public investment in the area. Consequently, the first-order conditions for the relevant variables can be obtained as follows:

$$\begin{array}{c} W_{t}^{2}=[(1-\tau_{t}^{q})Y_{t}^{2}P_{t}^{2}\left(1-x\right)]/[(L_{t}^{2}-1)(1+\tau_{t}^{1})] \end{array}$$ (32)

The following budget constraint can express the fiscal behavior of the government in Rural area:

$$\begin{array}{c} g_{t}^{2}=\Gamma_{t}T_{t}^{2} \end{array}$$ (33)

Here, $T_{t}^{2}$ represents the total tax revenue obtained by rural area through personal income tax, corporate income tax, value-added tax on capital elements, and value-added tax on labor elements. Γ denotes the tax revenue-sharing rules stipulated by the provincial government for rural area.

#### 4.1.3 Determination of tax revenue-sharing rules

For the provincial-level government, guided by the research of Zhou and Wu (2015), the influence of inter-regional economic development balance on the revenue-sharing ratio with lower-level governments is considered [32]. Thus, by constructing the relevant tax impact parameter Γ_*t*_, the proportion of local revenue-sharing is determined, resulting in the following tax revenue-sharing rule:

$$\begin{array}{c} \Gamma_{t}=(1-\frac{\left|y_{1}-y_{2}\right|}{y_{1}+y_{2}})*(\frac{Z_{i*}y_{1}}{Z_{1}*y_{1}+Z_{2}*y_{2}})*\eta \end{array}$$ (34)

Simultaneously, accounting for the proportional representation of urban and rural area, the behavioral equation for the provincial-level government can be defined as follows:

$$\begin{array}{c} (1-\Gamma_{t})*(\;T_{t}^{1}+T_{t}^{2})=g_{t} \end{array}$$ (35)

#### 4.1.4 Taxation and technological shocks

Introducing various tax shocks and technological shocks can be expressed as follows [33, 34].

Personal income tax shock:

$$\begin{array}{c} \tau_{t}^{s}={\left(\tau_{t-1}^{s}\right)}^{\rho \tau s}e^{\varepsilon \tau s} \end{array}$$ (36)

Corporate income tax shock:

$$\begin{array}{c} \tau_{t}^{s}={\left(\tau_{t-1}^{s}\right)}^{\rho \tau s}e^{\varepsilon \tau s} \end{array}$$ (37)

Value-added tax on labor elements shock:

$$\begin{array}{c} \tau_{t}^{s}={\left(\tau_{t-1}^{s}\right)}^{\rho \tau s}e^{\varepsilon \tau s} \end{array}$$ (38)

Value-added tax on capital elements shock:

$$\begin{array}{c} \tau_{t}^{1}={\left(\tau_{t-1}^{1}\right)}^{\rho \tau 1}e^{\varepsilon \tau 1} \end{array}$$ (39)

Technological shock:

$$\begin{array}{c} A_{t}=A_{t-1}e^{\varepsilon a} \end{array}$$ (40)

In summary, the model that incorporates the development of digital inclusive finance and intergovernmental relations at different levels is now constructed. The following steps involve parameter calibration and policy simulation analysis.

### 4.2 Parameter calibration

This study draws upon existing research findings to ensure that the empirical model aligns closely with the fundamental characteristics of current fiscal relations among regions in China. The calibration results are presented in Table 2.

**Table 2:** Calibration of key parameters.

Parameter Names	Symbol	Calibrated Value
Labor relative elasticity	*φ*	1.5
Consumption relative elasticity	*δ*	2
Spot discounting factor	*β*	0.8
Private capital output elasticity	*α*	1/3
Public capital output elasticity	*χ*	0.01
Provincial share of public investment in prefecture-level cities	S	0.01
Public capital depreciation rate	*ϕ*	0.025
Technology shock factor	*τ*_A_	0.6
Capital element value-added tax shock factor	*τ*_K_	0.6
Labor element value-added tax shock factor	*τ*_L_	0.6
Personal income tax shock factor	*τ*_s_	0.6
Bank convertibility ratio for private credit	λ_1_	0.4479
Bank convertibility ratio for government credit	λ_2_	0.2239
Entrepreneur survival discount factor	*θ*	0.98
Proportion of financial non-exclusionary regions	Z_1_	0.6
Proportion of financial exclusionary regions	Z_2_	0.4
Corporate income tax rate	*τ*_q_SS	0.15
Labor element value-added tax	*τ*_L_SS	0.3
Capital element value-added tax	*τ*_k_SS	0.1

#### 4.2.1 Household sector

Firstly, we calibrate the discount factor parameter by referencing the empirical research conducted by Wang et al. (2022), resulting in a calibrated value of 0.8 [35]. Secondly, we determine the intertemporal elasticity of substitution for consumption by adopting the methodology employed by Kang and Gong (2014) [36], resulting in a calibrated value of 2. Thirdly, we calculate the reciprocal of the intertemporal elasticity of labor supply by following the approach outlined by Zhang (2019), resulting in a calibrated value of 1.5 [37].

#### 4.2.2 Firm sector

This study adopts the methodology employed by Wang et al. (2021), who used Bayesian methods to fit Chinese enterprise data. The calibration of the elasticity of private capital *α* in production is conducted, yielding a calibrated value of 1/3. Additionally, the elasticity of public capital in production is calibrated with a value of 0.01 [35].

#### 4.2.3 Banking sector

This study draws upon the findings of Strobel (2018) to calibrate the private credit conversion ratio λ_1_ and the government credit conversion ratio λ_2_ in the banking sector, setting them at 0.4479 and 0.2239, respectively. Additionally, the entrepreneurial survival stickiness *θ* is specified at 0.98 [38].

#### 4.2.4 Government sector

In this study, we utilize the approach outlined by Liu et al. (2004) to calibrate the individual income tax rate at 0.15 using China’s 2008–2020 personal income tax data and defining it as a steady state [4, 39]. Simultaneously, following the provisions of the Chinese Corporate Income Tax Law, the corporate income tax rate is calibrated as *τ*_q_SS = 0.15. Drawing on the methodology of Ozili (2017), this paper utilizes the Social Accounting Matrix (SAM) to attempt to delineate value-added tax into labour factor value-added tax *τ*_*L*_SS and capital factor value-added tax *τ*_k_SS. These are estimated and calibrated from the SAM table, resulting in a tax rate of 0.3 for *τ*_*L*_SS and 0.1 for *τ*_k_SS [40].

#### 4.2.5 Financial sector

Building upon the statistical descriptive analysis of the dualistic urban-rural disparity in China presented earlier, this study, in the baseline model within a traditional financial context, establishes the proportions of urban and rural areas as 0.6 and 0.4, respectively. Furthermore, adjustments and counterfactual simulations are conducted during the policy simulation process based on the level of development in digital inclusive finance.

## 5 Dynamic simulation analysis and robustness testing

Following the general modelling procedure of DSGE models, entails constructing, deriving, and calibrating the mathematical model based on parameter inputs. Subsequently, we conduct random shock simulations and deterministic shock simulations.

### 5.1 Dynamic simulation analysis

#### 5.1.1 Stochastic shock simulation in a traditional financial context

In this section, stochastic simulations are employed to incorporate technological shocks and experiment with theoretical assumptions. We assume that the economic environment is subject to positive technological advancements. Grounded in the microeconomic behavior of the economy, we simulate dynamic response outcomes of key variables, including regional tax revenue-sharing ratios, government expenditure scales, regional tax revenue, household consumption, and output.

**Fig 2:**
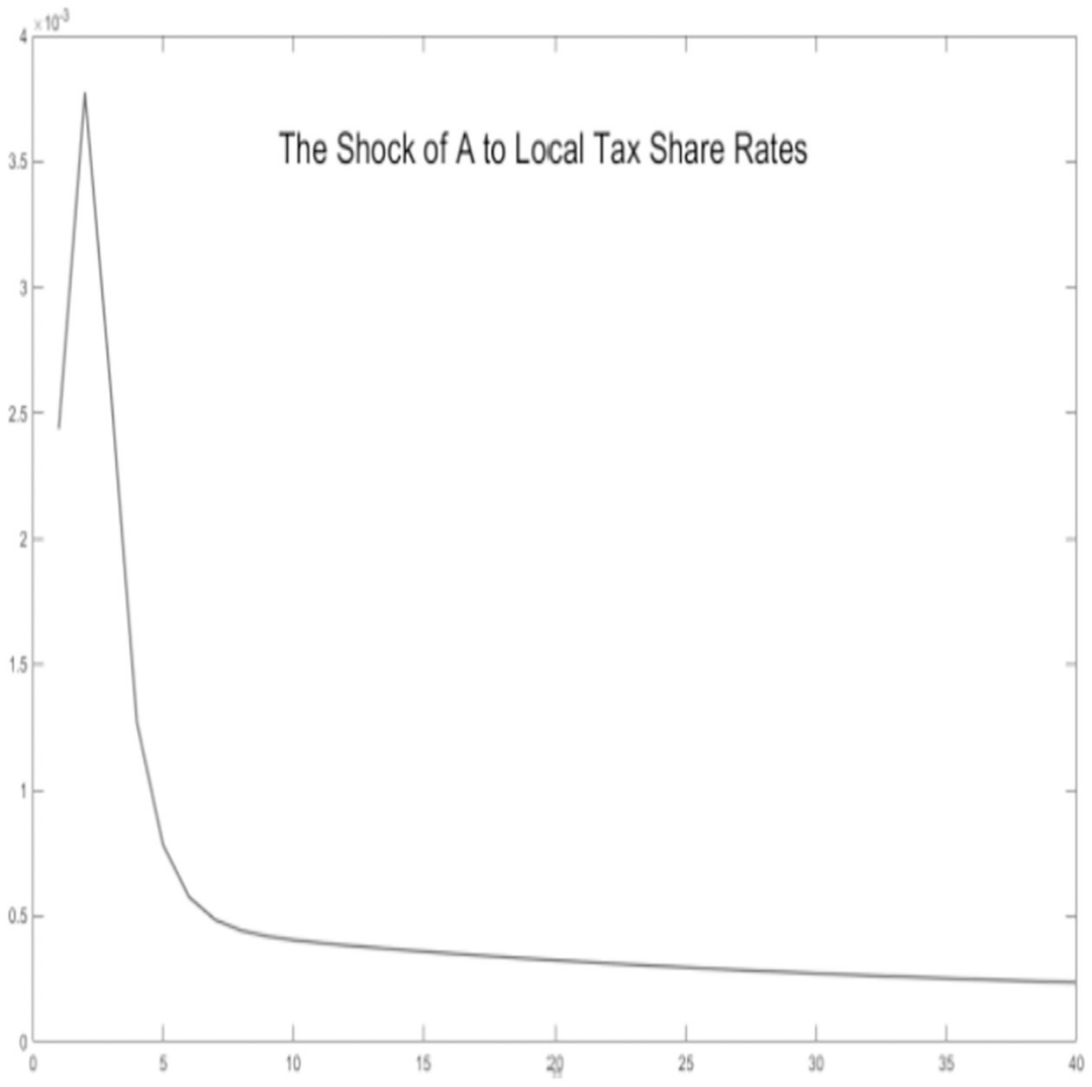
The shock of A to government spending.

**Fig 3:**
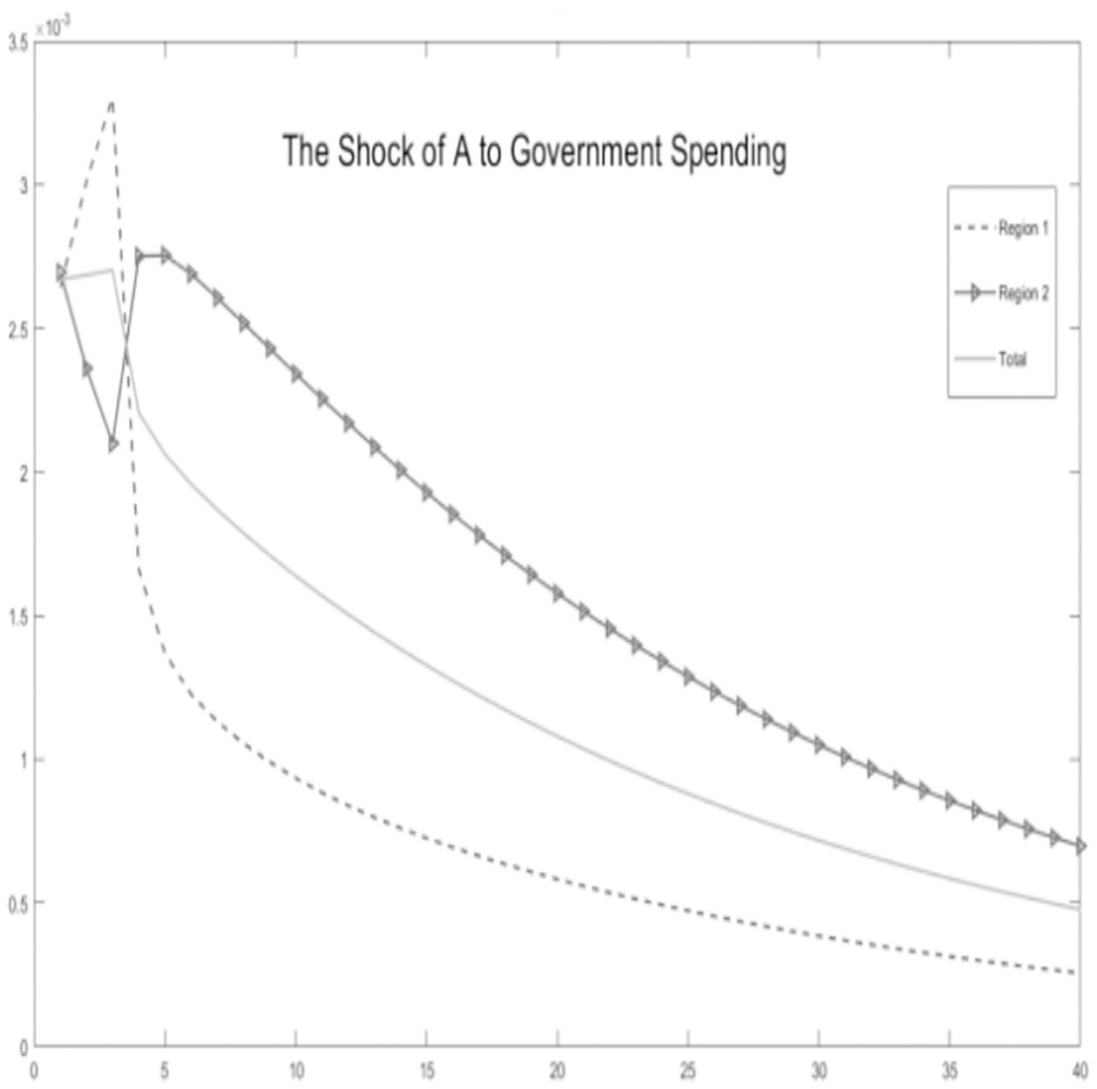
The shock of A to local tax share rates.

**Fig 4:**
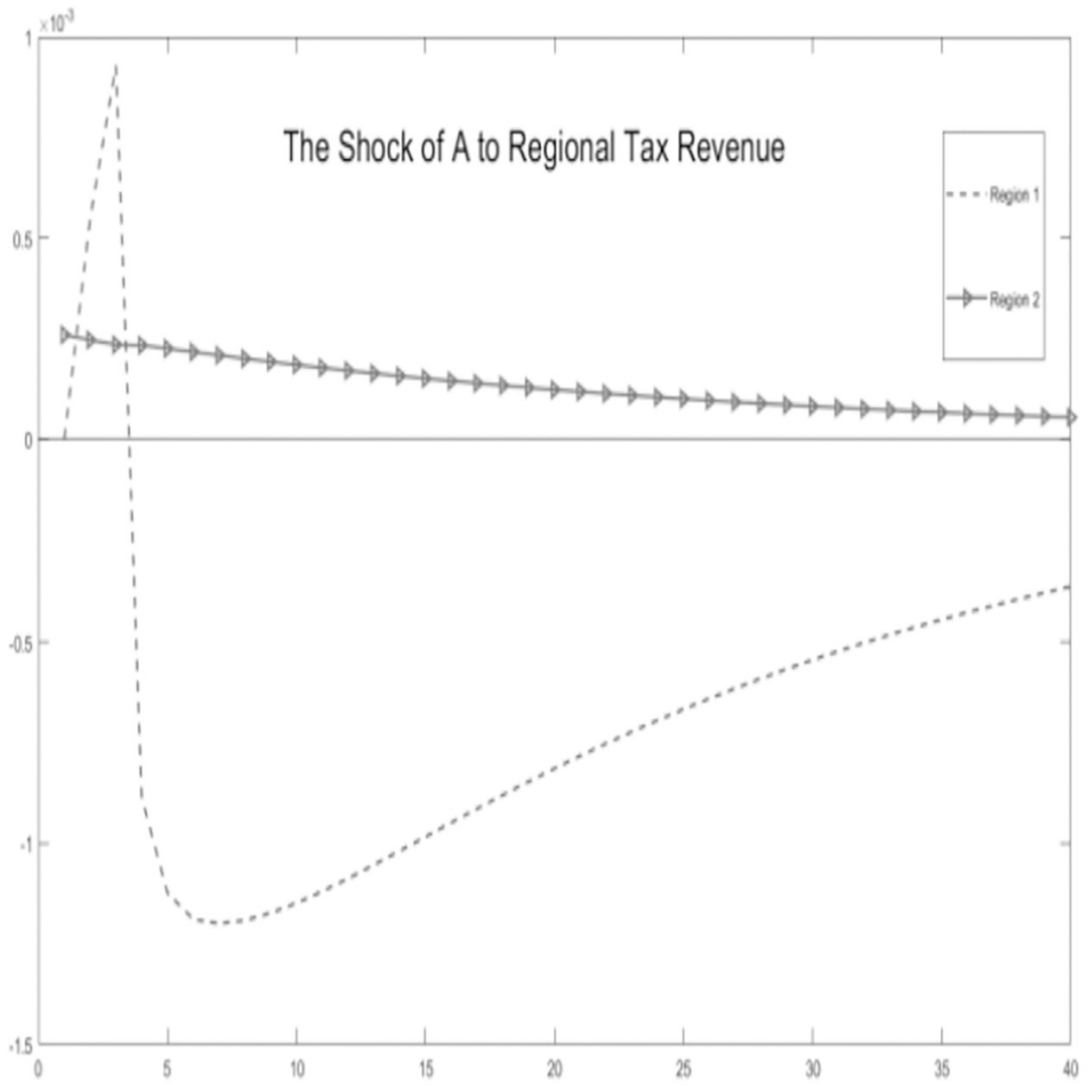
The shock of A to regional tax revenue.

**Fig 5:**
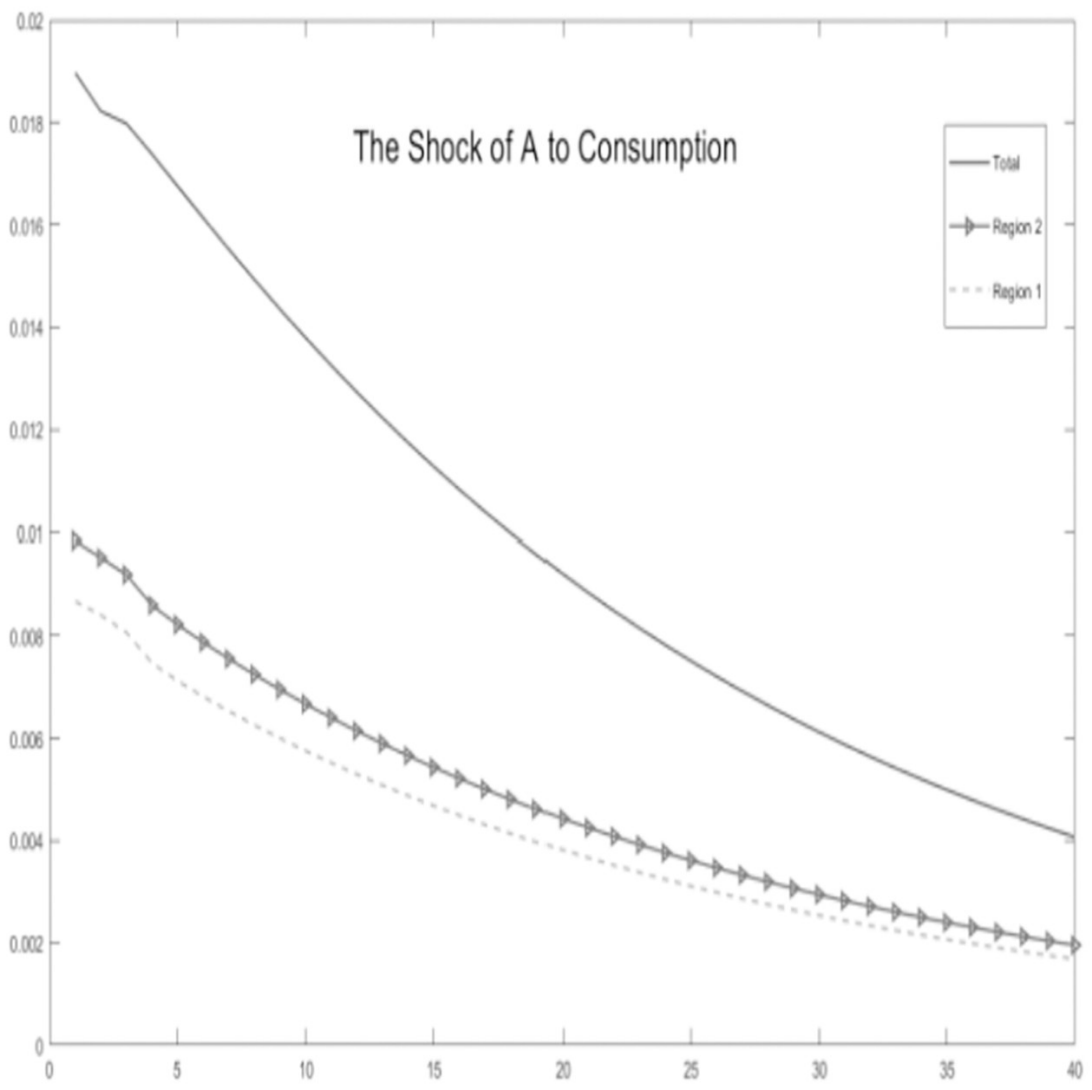
The shock of A to consumption.

**Fig 6:**
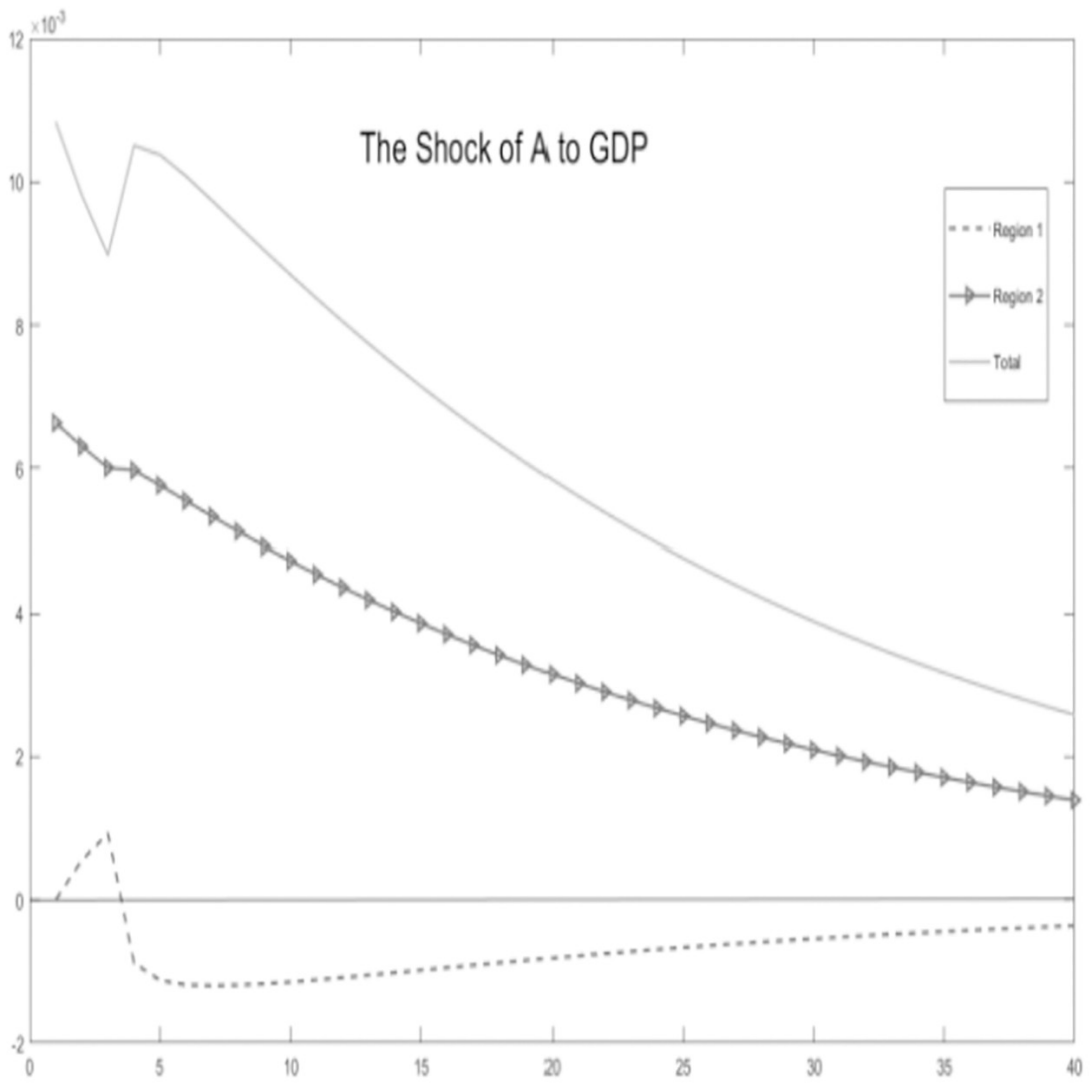
The shock of A to GDP.

Critical variables in the economic environment are influenced by the impact of technological progress, as illustrated Figs 2–6. It is observed that, for key macroeconomic variables such as regional output, consumption, the ratio of tax revenue, government expenditures, and tax revenues, the initial technological progress shock positively affects total production and resident consumption in both the urban area and the rural area. Furthermore, the output shock in the rural area significantly surpasses the output increase in the urban area. This discrepancy arises from the relatively saturated production level in the urban area. The rural area is still experiencing increasing returns to scale due to technological progress, making it more susceptible to achieving a total output increase.

Simultaneously, by examining impulse response graphs, it can be observed that, under the influence of positive technological advancements, the provincial-level government exhibits a positive shock response regarding the tax revenue-sharing ratio with local governments. This manifests as a decentralization of fiscal revenue authority and subsequent stabilization. Total government expenditure and regional government expenditures in both urban and rural areas initially exhibit positive shock responses. However, rural area shows a response that decreases before increasing in the later stages, in contrast to urban area. Microfoundations analysis suggests that, in the urban area characterized as a non-financial exclusionary area, government departments can utilize financial instruments such as bank credit leverage and debt issuance, thereby increasing local government fiscal expenditures.

Regarding the tax revenue for urban and rural areas, the initial impact of technological progress needs to be pronounced. However, in the later stages, urban area’s tax revenue experiences a rapid increase and stabilizes. This is because, although the tax revenue-sharing ratio of the lower-level government increases, its overall impact on the total tax revenue is minor. Conversely, in financially non-exclusionary areas, the judicious use of financial tools enables them to obtain higher returns on tax revenue through overall socio-economic growth.

In summary, the aforementioned variables show co-movement. Based on micro foundational logic, it is evident that, within the context of traditional urban-rural dual financial differentiation, an increase in the tax revenue-sharing ratio for municipal governments, coupled with socio-economic development represented by technological progress, leads to an augmentation of government fiscal expenditures at the local level while maintaining a balanced budget. This, in turn, results in an increase in overall socio-economic output and household consumption. The findings substantiate the impact of the pathway of traditional financial development on balancing regional economic development below the provincial level. This, in turn, influences the decentralization of fiscal and tax revenues at the provincial level and the scale of local government expenditures. This supports the hypothesis H1.

#### 5.1.2 Counterfactual deterministic simulation under the development of inclusive digital finance

Expanding on the baseline model that incorporates traditional financial sectors, this study further explores the impact of inclusive digital finance development on local fiscal decentralization and government behavior. Specifically, as stated in the theoretical assumptions, digital empowerment complements traditional finance, aiming for financial inclusivity. This enhances the financial accessibility and non-exclusivity of the rural area, achieving inclusive growth. Consequently, we maintain the assumption of Z1 as the proportion of non-exclusionary areas and Z2 as the proportion of financially exclusionary areas. Through counterfactual simulations based on the traditional financial model, we increase the proportion of Z1. This exploration aims to investigate the deepening effects of inclusive digital finance on the dynamics of revenue sharing and local government expenditure behavior.

The advantages and distinctive capabilities of using the DSGE model for counterfactual simulations are evident. Table 3 adjusted the proportion of digitally accessible regions compared to non-digitally accessible areas, ranging from 0% to 90%, and simulations were performed. The simulation results provide a more intuitive understanding.

**Table 3:** Counterfactual deterministic simulation results based on the development of digital inclusive finance.

Z1	Z2	Local tax revenue impact	Local government expenditure impact	Region 1 government expenditure impact	Region 2 government expenditure impact	Regional gross domestic product impact
0.1	0.9	0.025	0.1356	0.2252	0.0460	0.3422
0.2	0.8	0.0547↑	0.1505↑	0.2253↑	0.0757↑	0.3865↑
0.3	0.7	0.0895↑	0.1682↑	0.2255↑	0.1108↑	0.4480↑
0.4	0.6	0.1312↑	0.1894↑	0.2258↑	0.1530↑	0.5413↑
0.5	0.5	0.1824↑	0.2115↑	0.2264↑	0.2046↑	0.7074↑
0.6	0.4	0.2472↑	0.2490↑	0.2278↑	0.2702↑	1.1260↑
0.7	0.3	0.3543↑	0.3127↑	0.2455↑	0.3799↑	2.6640↑
0.8	0.2	0.4253↑	0.3355↑	0.2218↓	0.4493↑	-0.7589↓
0.9	0.1	0.5771↑	0.4134↑	0.2240↑	0.6027↑	-0.0727↑

As digital inclusive finance advances, increased financial accessibility allows remote rural areas to benefit from financial services. In the simulation process, assuming that the development of digital inclusive finance leads to an improvement in financial accessibility from 60% during the traditional financial period to 70%, it indicates an enhancement in urban-rural financial accessibility. Compared to the distribution of traditional finance and accompanying socio-economic development, the impact of the tax revenue-sharing ratio on provincial-level cities, compared to prefecture-level cities, increases by 43.33%. This implies that the development of digital inclusive finance will lead to balanced economic development within the provincial jurisdiction, resulting in fiscal decentralization represented by increased tax revenue sharing. This substantiates the validation of hypothesis H2a.

Government expenditure in the urban area increased by 7.78%, while in the rural area, it experienced a substantial rise of 40.59%. The overall local government expenditure increased by 25.58%, and the regional GDP witnessed a notable growth of 136.59%. This supports the establishment of hypothesis H2b. Moreover, compared to the urban area, the development of digital inclusive finance results in a more substantial expansion of local government expenditure in the rural area.

### 5.2 Robustness testing

#### 5.2.1 Traditional finance, fiscal decentralization, and local government expenditure

$$\begin{array}{c} {\text{govex}}_{i,t}=\alpha_{1}+\alpha_{2}\;{\text{trafin}}_{i,t}+\alpha_{3}\;{\text{controls}}_{i,t}+u_{it} \end{array}$$ (41)

$$\begin{array}{c} {\text{fiscde}}_{i,t}=\beta_{1}+\beta_{2}\;{\text{trafin}}_{i,t}+\beta_{3}\;{\text{controls}}_{i,t}+\theta_{\text{it}} \end{array}$$ (42)

Considering data availability and aligning with the existing digital inclusive finance index, we selected pertinent data from 2011 to 2020 for empirical analysis. (1) The dependent variable is local fiscal expenditure (govex), represented by the ratio of general public budgetary expenditure of local governments to local fiscal revenue. (2) The core independent variable, which reflects the level of local financial development (trafin), is expressed by the ratio of the loan scale of local commercial banks to the total scale of deposits and loans [41]. (3) The mediating variable is the degree of fiscal decentralization (fiscde), following the DGSE model construction method. It is represented by the ratio of provincial-level tax revenue to total tax revenue; a higher value indicates a more centralized fiscal power at the provincial level, suggesting lower fiscal decentralization. (4) Drawing from studies such as Mao et al. (2019), Christensen et al.,(2019), and Kinley et al. (2021) and on the “bank-government relationship” when selecting control variables, we include the regional urbanization rate (urblev), regional industrial structure (industr), regional per capita GDP (pgdp), government intervention degree (govin), fixed asset investment (fixinv), and urban-rural income gap (srcj) [42–45]. All the data are sourced from the CNRDS database, Wind database, and China Statistical Yearbook, and have been organized accordingly.

#### 5.2.2 Digital inclusive finance, fiscal decentralization, and local government expenditure

$$\begin{array}{c} {\text{govex}}_{i,t}=\gamma_{1}+\gamma_{2}\;{\text{digfin}}_{i,t}+\gamma_{3}\;{\text{controls}}_{i,t}+\delta_{it} \end{array}$$ (43)

$$\begin{array}{c} {\text{fiscde}}_{i,t}=\rho_{1}+\rho_{2}\;{\text{digfin}}_{i,t}+\rho_{3}\;{\text{controls}}_{i,t}+\sigma_{\text{it}} \end{array}$$ (44)

Where represents the level of development in digital inclusive finance, and the definitions of other variables are consistent with Eqs (41) and (42).

#### 5.2.3 Results analysis

Table 4 presents the descriptive statistics of the variables. Furthermore, to ensure the accuracy of the results in the regression model, a logarithmic transformation is applied to all variables.

**Table 4:** Provides the descriptive statistics of the variables.

Variable	Obs	Mean	Std	Min	Max
govex	300	0.7405	0.0907	0.5320	1.1207
fiscde	300	0.0178	0.0136	0.0014	0.0707
trafin	300	0.4443	0.0720	0.2757	0.8622
digfin	300	217.2461	96.9682	18.33	431.9276
fixinv	300	17619.87	12174.43	1435.58	55202.72
urblev	300	0.5901	0.1222	0.3504	0.8958
govin	300	0.01	0.0032	0.0021	0.0184
industr	300	12489.26	10967.51	540.18	62540.78
pgdp	300	56117.3	27475.45	16413	164889.5
srcj	300	2.5888	0.3784	1.85	3.67

Table 5 illustrates the influence of traditional financial development on local government expenditure in the first column and its impact on the fiscal decentralization of local governments below the provincial level in the second column. It is evident that local-level traditional financial development in China affects the fiscal decentralization of local governments below the provincial level, consequently influencing the magnitude of local government expenditure. More specifically, traditional financial development shows a notably positive impact on the magnitude of local government expenditure, signifying an increase in local government spending with the progression of traditional financial development. Additionally, in the context of fiscal decentralization from the provincial level to lower government tiers, traditional financial development mitigates the concentration of fiscal decentralization at the provincial level, resulting in the delegation of fiscal authority to municipal and county-level governments. Consequently, this triggers a rise in the overall government expenditure at the provincial level, establishing a close association in the local “bank-government relationship” [46–48]. The alignment of the conclusions with Hypothesis H1 and the resilience of the study findings are confirmed by the consistency observed in the counterfactual simulation results.

**Table 5:** Robustness testing outcome.

	(1)	(2)	(3)	(4)
	govex	fiscde	govex	fiscde
trafin	0.1245***	-0.4458***		
	(4.0506)	(-3.4757)		
digfin			0.0217***	-0.1116***
			(2.8210)	(-3.5322)
fixinv	0.0945***	-0.1869***	0.1009***	-0.2116***
	(7.0048)	(-3.3195)	(7.4039)	(-3.7760)
urblev	-0.1650**	0.3288	-0.1336*	0.1423
	(-2.4816)	(1.1846)	(-1.9263)	(0.4989)
govin	-0.1050***	0.3297***	-0.1121***	0.3674***
	(-6.3594)	(4.7858)	(-6.6059)	(5.2651)
industr	-0.1301***	1.0057***	-0.1413***	1.0448***
	(-10.3538)	(19.1786)	(-11.4100)	(20.5070)
pgdp	-0.0471*	-0.1828	-0.0366	-0.1962*
	(-1.7156)	(-1.5959)	(-1.3249)	(-1.7284)
srcj	0.0924**	-0.0848	0.0678	0.0329
	(2.2237)	(-0.4888)	(1.5902)	(0.1874)
cons	-0.0827	-8.2477***	-0.3635	-7.3086***
	(-0.2674)	(-6.3909)	(-1.1988)	(-5.8588)
N	300	300	300	300
R2	0.6780	0.8646	0.6690	0.8648

Note:

***, **, and * indicate statistical significance at the 1%, 5%, and 10% levels, respectively.

Column 3 of Table 5 illustrates the impact of digital inclusive financial development on local government expenditure in China, while Column 4 demonstrates its effect on the fiscal decentralization of local governments below the provincial level in China. It is evident that a higher level of digital inclusive financial development is conducive to local governments reducing fiscal centralization, thereby devolving financial power to municipal and county-level governments, resulting in a decline in fiscal authority for provincial-level governments, thus substantiating Hypothesis H2a. Additionally, it can be observed that as the level of digital inclusive financial development increases, the scale of local government expenditure also increases, indicating a direct positive effect of digital inclusive financial development on local government expenditure, validating Hypothesis H2b and further corroborating the robustness of the DSGE model simulation results in this study.

## 6 Conclusions and recommendations

The research above indicates that with the rapid advancement of digital finance, there is an improvement in digital financial accessibility. This contributes to the reduction of urban-rural income disparities and strengthens balanced economic development across regions. Building upon this foundation, fiscal decentralization, represented by the shift in tax revenue sharing to levels below the provincial, facilitates the expansion of fiscal expenditures by municipal governments. This stimulates short-term economic growth. However, it is essential to note that in the long term, with the deepening development of digital inclusive finance, the decentralization of fiscal powers and the expansion of local government expenditures may not only crowd out private investment and household consumption but also lead to sustained government expenditure and negative externalities. This, in turn, results in a deceleration of regional economic growth, impeding regional economic development. Based on the research findings above, this paper proposes the following recommendations:

Encouraging the development of digital inclusive finance. Firstly, nations should proactively seize the opportunities presented by the global development of digital finance, fostering the establishment of financial infrastructure in remote and underdeveloped areas such as rural regions. Secondly, governments worldwide should tailor regulatory measures and policy choices based on their unique developmental contexts. Countries prioritizing digital finance development should initiate transformative adjustments, refining legal and regulatory frameworks concerning digital taxation, among other aspects. For nations with a slower pace of digital finance development, leveraging digital financial technologies and tools is crucial to overcoming information asymmetry among governments. Strengthening communication and collaboration with countries experiencing positive momentum in digital finance development is essential for identifying and addressing issues promptly. Thirdly, harnessing the opportunities provided by digital inclusive finance mechanisms to offer more avenues for growth can significantly contribute to fundamentally narrowing income disparities worldwide. This approach facilitates balanced development across regions, thereby promoting global economic equilibrium.

Optimizing the fiscal decentralization policy system. Firstly, governments should increase financial support for agriculture, expand public investment in rural areas, effectively improve rural infrastructure construction, enhance rural production and living standards, invest in agricultural technology, and raise the incomes of people living in rural areas. Countries should continue to optimize the governance model of fiscal decentralization, clarifying the rights and responsibilities of the government in promoting regional economic growth. Efforts should be made to address the adverse impacts of the fiscal decentralization system on regional economic development. Secondly, governments should make good use of fiscal decentralization to promote regional economic growth. They should play a synergistic role in providing financial support for agriculture and promoting digital financial inclusion. Financial support for agriculture can be used to leverage the depth of digital financial inclusion in rural areas, reduce production costs and financial disincentives, narrow the income gap between urban and rural areas, reform and improve the transfer payment system, and achieve the basic equalization of inter-regional public services. This will help implement national macroeconomic policies while empowering people to make the best use of their resources. In addition to promoting the implementation of national macro-policies, it also empowers local innovative activities. Thirdly, to improve the performance appraisal system of local governments, countries should incorporate digital financial inclusion indicators into the performance appraisal system. This should be based on the redivision of the scope of authority to guide the flow of government funds and stimulate the role of digital finance in promoting economic growth.

## Supporting information

S1 File (MOD)

S1 Data (DTA)

## References

[1] Zeng Z. Does financial decentralization create financial risk? Advances in Economics and Management Research, 2023; 6, 597–597. doi: 10.56028/aemr.6.1.597.2023

[2] Reinhart CM, Rogoff KS. From financial crash to debt crisis. American Economic Review, 2011; 5, 1676–1706. doi: 10.1257/aer.101.5.1676

[3] Acharya V, Drechsler I, Schnabl PA. Pyrrhic victory? Bank bailouts and sovereign credit risk. Finance, 2014; 6, 2689–2739. doi: 10.1111/jofi.12206

[4] Liu C, Moldogaziev TT, Mikesell JL. Corruption and state and local government debt expansion. Public Administration Review, 2017; 5, 681–690. doi: 10.1111/puar.12711

[5] Gadenne L, Singhal M. Decentralization in developing economies. Annual Review of Economics, 2014; 6, 581–604. doi: 10.1146/annurev-economics-080213-040833

[6] Li J, Li B. Digital inclusive finance and urban innovation: Evidence from China. Review of Development Economics, 2022; 26, 1010–1034. doi: 10.1111/rode.12846

[7] Jack W, Ray A, Suri T. Transaction networks: Evidence from mobile money in Kenya. American Economic Review, 2013; 103, 356–361. doi: 10.1257/aer.103.3.356

[8] Yu C, Jia N, Li W, Wu R. Digital inclusive finance and rural consumption structure–evidence from Peking University digital inclusive financial index and China household finance survey. China Agricultural Economic Review, 2022; 14, 165–183. doi: 10.1108/CAER-10-2020-0255

[9] Simionescu M, Cifuentes‐Faura J. Analysing public debt in the Mexican states: Spatial convergence, regional drivers and policy recommendations. Papers in Regional Science, 2023; 102, 737–760. doi: 10.1111/pirs.12748

[10] Ariyanto D, Soejono F, Dewi SP. Digital economy and financial inclusion. Environmental Treatment Techniques, 2020; 1, 241–245. Available from: http://eprints.ukmc.ac.id/id/eprint/3583

[11] Molotok I. Does fiscal decentralization influence on management efficiency of country innovative development? Marketing and Management of Innovations, 2020; 1, 54–62. Available from: https://essuir.sumdu.edu.ua/handle/123456789/77085 doi: 10.21272/mmi.2020.1-04

[12] Gillman M. Income tax evasion: Tax elasticity, welfare, and revenue. International Tax and Public Finance, 2021; 28, 533–566. doi: 10.1007/s10797-020-09632-3

[13] Jin Y, Rider M. Does fiscal decentralization promote economic growth? An empirical approach to the study of China and India. Public Budgeting, Accounting & Financial Management, 2022; 34, 146–167. doi: 10.1108/JPBAFM-11-2019-0174

[14] Cifuentes-Faura J, Fülöp MT, Topor DI. Financial autonomy in Spanish local governments: Empirical evidence of beta and sigma convergence. International Review of Administrative Sciences, 2023. doi: 10.1177/00208523231209655

[15] Ding Y, McQuoid A, Karayalcin C. Fiscal decentralization, fiscal reform, and economic growth in China. China Economic Review, 2019; 53, 152–167. doi: 10.1016/j.chieco.2018.08.005

[16] Kim Y, Su L, Wang Z, Wu H. The effect of trade secrets law on stock price synchronicity: Evidence from the inevitable disclosure doctrine. Accounting Review, 2021; 96, 325–348. doi: 10.2308/tar-2017-0425

[17] Brennan HG, Buchanan JM. The Power to Tax: Analytical Foundations of a Fiscal Constitution. Liberty Fund, 2000. Available from: https://www.jstor.org/stable/2724932

[18] Oates WE. Searching for Leviathan: An empirical study. American Economic Review, 1985; 75, 748–757. Available from: https://www.jstor.org/stable/1821352

[19] Stein E. Fiscal decentralization and government size in Latin America. Applied Economics, 1999; 2, 357–391. doi: 10.1080/15140326.1999.12040543

[20] Rodden J. The dilemma of fiscal federalism: Grants and fiscal performance around the world. American Journal of Political Science, 2002; 670–687. doi: 10.2307/3088407

[21] Baskaran T. On the link between fiscal decentralization and public debt in OECD countries. Public Choice, 2010; 145, 351–378. doi: 10.1007/s11127-009-9570-4

[22] Pasichnyi M, Kaneva T, Ruban M, Nepytaliuk A. The impact of fiscal decentralization on economic development. Investment Management and Financial Innovations, 2019; 16. Available from: https://ssrn.com/abstract=3430955 doi: 10.21511/imfi.16(3).2019.04

[23] Gemmell N, Kneller R, Sanz I. Fiscal decentralization and economic growth: spending versus revenue decentralization. Economic Inquiry, 2013; 51, 1915–1931. doi: 10.1111/j.1465-7295.2012.00508.x

[24] Cifuentes-Faura J, Benito B, Guillamón MD, Faura-Martínez Ú. Relationship between transparency and efficiency in municipal governments: Several non-parametric approaches. Public Performance & Management Review, 2023; 46(1), 193–224. doi: 10.1080/15309576.2022.2123007

[25] Wu M, Zhou LA, Shi G. Typification of Tax Revenue Sharing by Chinese County Governments: Estimation and Analysis Based on Unique Data. Finance and Trade Economics, 2023; 44, 5–20. Available from: https://www.cnki.net/KCMS/detail/detail.aspx?dbcode=CJFD&dbname=CJFDLAST2023&filename=CMJJ202304001&uniplatform

[26] Hasan MM, Lu YJ, Khan S. Promoting China’s inclusive finance through digital financial services. Global Business Review, 2022; 4, 984–1006. doi: 10.1177/0972150919895348

[27] Guo F, Wang JY, Wang F, Kong T, Zhang X, Cheng ZY. Measuring the Development of Digital Inclusive Finance in China: Index Construction and Spatial Characteristics. Economic Research Journal, 2020; 19, 1401–1418. doi: 10.13821/j.cnki.ceq.2020.03.12

[28] Qiao M, Ding S, Liu Y. Fiscal decentralization and government size: The role of democracy. European Journal of Political Economy, 2019; 59, 316–330. doi: 10.1016/j.ejpoleco.2019.04.002

[29] Martínez‐Vázquez J, Lago‐Peñas S, Sacchi A. The impact of fiscal decentralization: A survey. Economic Surveys, 2017; 31, 1095–1129. doi: 10.1111/joes.12182

[30] Ramey VA. Macroeconomic shocks and their propagation. Handbook of Macroeconomics, 2016; 2: 71–162. doi: 10.1016/bs.hesmac.2016.03.003

[31] Mertens K, Ravn MO. The dynamic effects of personal and corporate income tax changes in the United States. American Economic Review, 2013; 103, 1212–1247. doi: 10.1257/aer.103.4.1212

[32] Zhou LA, Wu M. Tax Revenue Sharing Among Multilevel Governments Below the Provincial Level: Empirical Facts and Explanations. Financial Research, 2015; 10, 64–80. Available from: https://www.cnki.net/KCMS/detail/detail.aspx?dbcode=CJFD&dbname=CJFDLAST2016&filename=JRYJ201510005&uniplatform

[33] Ding L, Wu Y, Long J. Incentive effect of tax preferences towards the technological innovation of enterprises—Based on China’s GEM listed companies. PLOS ONE, 2023; 18, e0282692. doi: 10.1371/journal.pone.0282692 37058543 PMC10104326

[34] Attanasio OP, Paiella M. Intertemporal consumption choices, transaction costs and limited participation in financial markets: reconciling data and theory. Journal of Applied Econometrics, 2011; 26, 322–343. doi: 10.1002/jae.1154

[35] Wang X, Li J, Zhang G. Mixed monetary–fiscal policies and macroeconomic fluctuations: An analysis based on the dynamic stochastic general equilibrium model. China and World Economy, 2022; 2, 167–196. doi: 10.1111/cwe.12414

[36] Kang L, Gong LT. Financial Frictions, Bank Net Assets, and the Transmission of the International Economic Crisis: An Analysis Based on a Multi-Sector DSGE Model. Economic Research, 2014; 49, 147–159. Available from: https://www.cnki.net/KCMS/detail/detail.aspx?dbcode=CJFD&dbname=CJFDLAST2015&filename=JJYJ201405012&uniplatform

[37] Zhang Y. The Macroeconomic Effects of Structural Tax Cuts and Expanded Government Expenditure. Economic and Management Research, 2019; 40, 20–38. Available from: https://www.cnki.net/KCMS/detail/detail.aspx?dbcode=CJFD&dbname=CJFDLAST2019&filename=JJYG201909002&uniplatform

[38] Strobel F. The government spending multiplier, fiscal stress and the zero lower bound. IMFS Working Paper Series, 2018. Available from: https://www.econstor.eu/handle/10419/183601

[39] Wang WF, Ai F, Wang ZQ. Adjustments to the Central-Local Fiscal Relationship and Macroeconomic Fluctuations. Journal of Finance and Economics, 2021; 2, 13–23. doi: 10.13762/j.cnki.cjlc.2021.02.002

[40] Ozili PK. Impact of Digital Finance on Financial Inclusion and Stability. Borsa Istanbul Review, 2017; 4, 329–340. doi: 10.1016/j.bir.2017.12.003

[41] Chen YR, Xiong DP. Inclusive growth effects of urban-rural financial development in China: An empirical analysis based on provincial panel data. Journal of Yunnan University of Finance and Economics, 2021; 37, 63–79. doi: 10.16537/j.cnki.jynufe.000728

[42] Mao J, Liu P, Lv BY. Institutional foundations of local public debt growth: Balancing fiscal and financial perspectives. China Social Science, 2019; 9, 45–67+205. Available from: https://www.cnki.net/KCMS/detail/detail.aspx?dbcode=CJFD&dbname=CJFDLAST2019&filename=ZSHK201909003&uniplatform

[43] Christensen RC, Hearson M. The New Politics of Global Tax Governance: Taking Stock a Decade after the Financial Crisis. Review of International Political Economy, 2019; 5, 106. doi: 10.1080/09692290.2019.1625802

[44] Kinley R, Cutajar MZ, DeBoer Y, Figueres C. Beyond good intentions, to urgent action: Former UNFCCC leaders take stock of thirty years of international climate change negotiations. Climate Policy, 2021; 5, 593–603. doi: 10.1080/14693062.2020.1860567

[45] Bradbury JC, Stephenson EF. Local government structure and public expenditures. Public Choice, 2003; 115, 185–198. Available from: https://link.springer.com/article/10.1023/A:1022857028836.

[46] Arif MZ, Chishti MZ. Analyzing the Effectiveness of Fiscal Decentralization in Economic Growth: The Role of Institutions. *Iranian Economic Review*, 2022; 26, 325–341. doi: 10.22059/IER.2022.88167

[47] Gu S, Zhang Q. The “heterogeneous” effect of government grants on bank lending. *Plos one*, 2023; 18, e0289375. doi: 10.1371/journal.pone.0289375 38079391 PMC10712854

[48] Ozili PK, Ademiju A, Rachid S. Impact of financial inclusion on economic growth: Review of existing literature and directions for future research. International Journal of Social Economics, 2023; 50, 1105–1122. doi: 10.1108/IJSE-05-2022-0339

